# Transcription factor YY1 inhibits the expression of THY1 to promote interstitial pulmonary fibrosis by activating the HSF1/miR-214 axis

**DOI:** 10.18632/aging.103142

**Published:** 2020-05-12

**Authors:** Lin Chen, Yang Yang, Xiaying Peng, Haiying Yan, Xin Zhang, Lin Yin, Hua Yu

**Affiliations:** 1 Department of Respiratory and Critical Care Medicine, Sichuan Academy of Medical Sciences and Sichuan Provincial People's Hospital, University of Electronic Science and Technology of China, Chengdu 611731, P.R. China; 2 Department of Laboratory Medicine, Sichuan Academy of Medical Sciences and Sichuan Provincial People's Hospital, University of Electronic Science and Technology of China, Chengdu 611731, P.R. China

**Keywords:** Yin Yang 1, heat shock transcription factor 1, microRNA-214, THY1, interstitial pulmonary fibrosis

## Abstract

Interstitial pulmonary fibrosis (IPF) is a progressive disease of diverse etiology manifesting with proliferation of lung fibroblasts and accumulation of extracellular matrix deposition in pulmonary interstitium. Recent studies show aberrant expression of mRNAs and microRNAs (miRNAs) in human embryonic pulmonary fibroblasts (HEPFs). In this study, we investigated effects of the YY1/HSF1/miR-214/THY1 axis on the functions of HEPFs and IPF. Loss- and gain-of-function tests were conducted to identify roles of YY1, HSF1, miR-214, and THY1 in IPF. As determined by RT-qPCR or western blot assay, silencing YY1 down-regulated HSF1 expression and attenuated the expression of pro-proliferative and fibrosis markers in HEPFs. Meanwhile, viability of HEPFs was impeded by YY1 knockdown. The binding relationship between miR-214 and THY1 was verified using dual-luciferase reporter assay. In HEPFs, down-regulation of HSF1 reduced miR-214 expression to repress proliferation and fibrogenic transformation of HEPFs, while inhibition of miR-214 expression could restrain the fibrogenic transformation property of HEPFs by up-regulating THY1. Subsequently, IPF model in mice was induced by bleomycin treatment. These animal experiments validated the protective effects of YY1 knockdown against IPF-induced lung pathological manifestations, which could be reversed by THY1 knockdown. Our study demonstrates the important involvement of YY1/HSF1/miR-214/THY1 axis in the development of IPF.

## INTRODUCTION

Idiopathic pulmonary fibrosis (IPF), characterized by the histopathological pattern of usual interstitial pneumonia, is the most common type of idiopathic interstitial pneumonia [1]. Micro-injuries that initiate abnormal epithelial-fibroblast communication, and induce matrix-producing myofibroblasts, are considered to contribute to the pathogenesis of IPF [1]. The myofibroblasts can originate from various sources, such as epithelial to mesenchymal transition, circulating fibrocytes, and endothelial mesenchymal transition [1]. The extracellular matrix components produced by activated myofibroblasts have been reported to further contribute to fibroblast activation, and moreover, the altered extracellular matrix itself promotes fibrosis [2]. However, the precise mechanism by which fibroblasts are activated and contribute to the pathogenesis of IPF remains unclear.

Yin-Yang 1 (YY1) is a transcription factor that plays a critical role in modulating the gene transcription [3]. Up-regulated YY1 has been reported in multiple types of cancers and has been associated with poor prognosis [3]. Interestingly, increased YY1 expression has also been observed in lung tissues from patients with IPF and murine models of lung fibrosis [4] suggesting its importance in the pathogenesis of IPF.

Heat shock factor-1 (HSF-1) is a master regulator controlling the expression of HSP genes, which counteract cellular stress and regulate disease states and aging [5]. It has also been reported that YY1 can directly activate the transcription of HSF-1 [6]. More importantly, a recent study has shown that HSF-1 facilitates the progression of IPF by up-regulating the expression of fibroblast growth factor-1 in the wounded lung epithelium [7]. Interestingly, HSF-1 has also been reported to mediate the transcription of the non-coding mRNA miR-214 [8]. Notably, miR-214-3p has been demonstrated to promote lung fibrosis by positively regulating the fibroblast-to-myofibroblasts transition [9].

Thymocyte differentiation antigen-1 (THY-1), also known as CD90 (Cluster of Differentiation 90), is a highly conserved glycoprotein that has been demonstrated to be a cancer biomarker [10]. An increasing body of evidence indicates that THY-1 can attenuate IPF [11, 12]. However, the mechanism by which THY-1 is involved in the pathogenesis of IPF remains unclear. In this study, we have identified the YY1-HSF1-miR-214-THY-1 axis, as a novel pathway playing a key role in the development and progression of IPF.

## RESULTS

### Involvement of YY1 and HSF1 in proliferation and fibrogenic transformation of pulmonary fibroblasts

**Figure 1:**
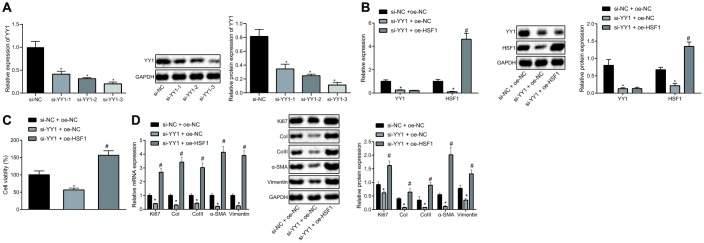
**HSF1 knockdown may suppress the proliferation and fibrogenic transformation of HEPFs.** (**A**) The YY1 expression in HEPFs transfected with three si-YY1 sequences determined at mRNA and protein levels by RT-qPCR and western blot analysis, respectively. *, *p* < 0.05 *vs.* cells transfected with si-NC. (**B**) The expression of YY1 and HSF1 in the HEPFs co-transfected with si-YY1/si-NC and oe-HSF1/oe-NC measured at mRNA and protein levels by RT-qPCR and western blot analysis, respectively. *, *p* < 0.05 *vs.* cells treated with si-NC and oe-NC. (**C**) The viability of HEPFs co-transfected with si-YY1/si-NC and oe-HSF1/oe-NC assessed by CCK8 experiment. *, *p* < 0.05 *vs.* cells after treatment of si-NC and oe-NC, #, *p* < 0.05 *vs.* cells transfected with si-YY1 and oe-NC. (**D**) The expression of cell proliferation marker Ki67 and fibrosis biomarkers CoI, CoIII, α-SMA and vimentin in the HEPFs co-transfected with si-YY1/si-NC and oe-HSF1/oe-NC measured at mRNA and protein levels by RT-qPCR and western blot analysis, respectively. *, *p* < 0.05 *vs.* cells co-transfected with si-NC and oe-NC, #, *p* < 0.05 *vs.* cells co-transfected with si-YY1 and oe-NC. Statistical data were measurement data, and presented as mean ± standard deviation. Unpaired *t*-test was used for comparison between the two groups, and one-way ANOVA with Tukey's post *hoc test* was employed for comparisons among multiple groups. The experiment was independently repeated three times.

To explore the correlation between YY1 and HSF1, siRNAs targeting YY1 (si-YY1) were designed to knock down YY1 and HSF1 overexpression plasmid (oe-HSF1) was co-transfected to the HEPFs treated with si-YY1. si-YY1-3 with the best silencing efficiency was firstly determined by reverse transcription-quantitative polymerase chain reaction (RT-qPCR) and western blot analysis (Figure 1A). The overexpression efficiency of HSF1 attained the requirements for further experiments (Figure 1B). Besides, YY1 silencing reduced the expression of HSF1, suggesting a positive regulatory effect of YY1 on HSF1. In the process of pulmonary fibrosis, a critical phenomenon is the proliferation of fibroblasts and their fibrogenic transformation into myofibroblasts. Therefore, the viability of human embryonic pulmonary fibroblasts (HEPFs) was assessed by CCK8 experiments after YY1 silencing and/or HSF1 overexpression (Figure 1C), which showed that silencing YY1 could inhibit the viability of HEPFs, while overexpression of HSF1 had the opposite effect. Subsequently, the expression of cell proliferation marker Ki67 and fibrosis biomarkers CoI, CoIII, α-SMA and vimentin was measured by RT-qPCR and western blot analysis (Figure 1D). The results indicated that silencing YY1 could suppress the expression of Ki67 and that of fibrosis biomarkers, while overexpression of HSF1 had opposite effects. Hence, YY1 silencing potentially impeded the proliferation and fibrogenic transformation of HEPFs by inhibiting HSF1.

### HSF1 promotes the proliferation and fibrogenic transformation of HEPFs by positively regulating miR-214

**Figure 2:**
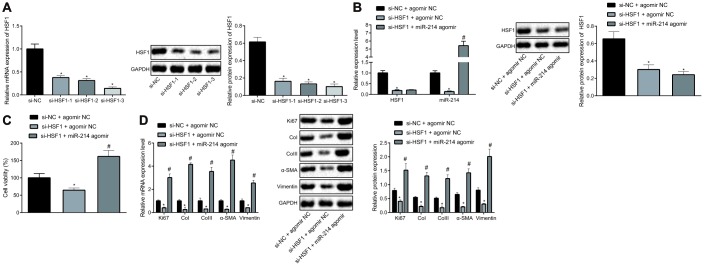
**HSF1 positively regulates the expression of miR-214 to affect the proliferation and fibrogenic transformation of HEPFs.** (**A**) The HSF1 expression in HEPFs transfected with three si-HSF1 sequences determined at mRNA and protein levels by RT-qPCR and western blot analysis, respectively. *, *p* < 0.05 *vs.* cells transfected with si-NC. (**B**) The expression of miR-214 and mRNA and protein levels of HSF1 in the HEPFs co-transfected with si-HSF1/si-NC and miR-214 agomir/agomir-NC measured by RT-qPCR and western blot analysis. *, *p* < 0.05 *vs.* cells co-transfected with si-NC and agomir NC, #, *p* < 0.05 *vs.* cells transfected with si-HSF1 and agomir NC. (**C**) The viability of HEPFs co-transfected with si-HSF1/si-NC and miR-214 agomir/agomir-NC assessed by CCK8 experiment. *, *p* < 0.05 *vs.* cells co-transfected with si-NC and agomir NC, #, *p* < 0.05 *vs.* cells co-transfected with si-HSF1 and agomir NC. (**D**) The mRNA and protein levels of cell proliferation marker Ki67 and fibrosis biomarkers CoI, CoIII, α-SMA and vimentin in the HEPFs co-transfected with si-HSF1/si-NC and miR-214 agomir/agomir-NC assessed by qRT-PCR and western blot analysis, respectively. *, *p* < 0.05 *vs.* cells co-transfected with si-NC and agomir NC, #, *p* < 0.05 *vs.* cells co-transfected with si-HSF1 and agomir NC. Statistical data were measurement data, and presented as mean ± standard deviation. Unpaired *t*-test was used for comparison between the two groups, and one-way ANOVA with Tukey's *post hoc* test was employed for comparisons among multiple groups. The experiment was independently repeated three times.

With an attempt to further explore the interaction between HSF1 and miR-214, HSF1 was silenced in HEPFs with siRNAs and miR-214 was overexpressed using miR-214 agomir. si-HSF1-3 proved to have the best silencing efficiency as shown by RT-qPCR and western blot analysis (Figure 2A). Then expression of miR-214 and HSF1 mRNA was measured by RT-qPCR, and protein expression of HSF1 by western blot analysis (Figure 2B). The results showed that the overexpression and silencing efficiency met the requirements for further experiments and that HSF1 silencing contributed to down-regulation of miR-214 expression. The viability of HEPFs in response to gain-/loss-of-function of HSF1 and miR-214 was assessed by CCK8 experiments (Figure 2C), which showed that silencing HSF1 could inhibit the viability of HEPFs, while overexpression of miR-214 could reverse this inhibitory effect on cell viability. The expression of cell proliferation marker Ki67 and fibrosis biomarkers CoI, CoIII, α-SMA and vimentin was measured by RT-qPCR and western blot analysis (Figure 2D), showing that silencing HSF1 reduced the expression of Ki67 and fibrosis biomarkers. However, these reductions were counteracted by overexpression of miR-214. In brief, HSF1 upregulated the expression of miR-214. Silencing HSF1 could suppress the fibrogenic transformation of HEPFs into myofibroblasts through down-regulation of miR-214.

### Inhibition of miR-214 impedes the proliferation and fibrogenic transformation of HEPFs by elevating THY1

**Figure 3:**
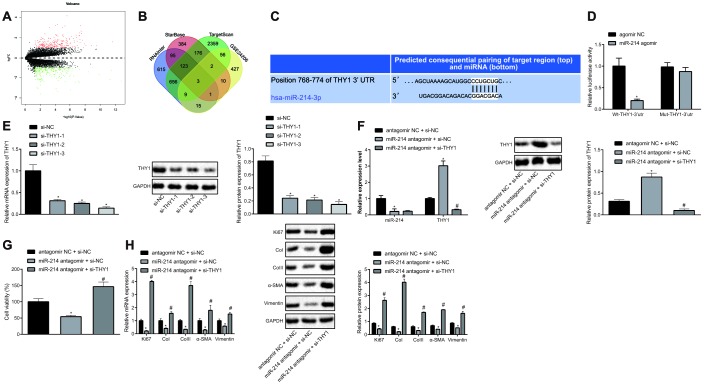
**Inhibition of miR-214 up-regulates THY1 to retard the proliferation and fibrogenic transformation of HEPFs.** (**A**) Volcano map of the DEGs in the IPF obtained from microarray GSE24206, the abscissa represented the different log10 *p* value, the ordinate indicated logFC. Each dot in the map represented a gene, where red dots represent the up-regulated gene, and green dots represent the down-regulated gene in the IPF sample; (**B**) Venn diagram showing target genes of miR-214 predicted by TargetScan database, StarBase database and RNAInter database, respectively and DEGs in the IPF obtained from microarray GSE24206 and the intersection gene among those four sets; (**C**) The possible binding sites of miR-214 and THY1 predicted by TargetScan database; (**D**) The binding region between miR-214 and THY1 obtained from TargetScan database, and their relationship was verified by dual-luciferase reporter assay using recombinant Wt-THY1-3’UTR and Mut-THY1-3’UTR. *, *p* < 0.05 *vs.* cells transfected with agomir NC; (**E**) The THY1 mRNA and protein levels in HEPFs transfected with three si-THY1 sequences determined by RT-qPCR and western blot analysis, respectively. *, *p* < 0.05 *vs.* cells transfected with si-NC; (**F**) The expression of miR-214 and mRNA expression of THY1 measured by RT-qPCR, and the protein expression of THY1 assessed by western blot analysis in the HEPFs co-transfected with si-THY1/si-NC and miR-214 antagomir/antagomir-NC. *, *p* < 0.05 *vs.* cells co-transfected with si-NC and agomir NC, #, *p* < 0.05 *vs.* cells co-transfected with miR-214 antagomir and si-NC; (**G**) The viability of HEPFs co-transfected with si-THY1/si-NC and miR-214 antagomir/antagomir-NC evaluated by CCK8 experiment. *, *p* < 0.05 *vs.* cells co-transfected with antagomir NC and si-NC, #, *p* < 0.05 *vs.* cells co-transfected with miR-214 antagomir and si-NC; (**H**) The mRNA and protein levels of cell proliferation marker Ki67 and fibrosis biomarkers CoI, CoIII, α-SMA and vimentin in the HEPFs co-transfected with si-THY1/si-NC and miR-214 antagomir/antagomir-NC assessed by qRT-PCR and western blot analysis. *, *p* < 0.05 *vs.* cells co-transfected with antagomir NC and si-NC, #, *p* < 0.05 *vs.* cells co-transfected with miR-214 antagomir and si-NC. Statistical data were measurement data, and presented as mean ± standard deviation. Unpaired *t*-test was used for comparison between the two groups, and one-way ANOVA with Tukey's *post hoc* test was employed for comparisons among multiple groups. The experiment was independently repeated three times.

Further, we analyzed the GSE24206 microarray to screen out the differentially expressed genes (DEGs) in IPF, which yielded 523 DEGs, of which 252 were up-regulated genes and 271 were down-regulated genes (Figure 3A). The target genes of miR-214 were predicted by the TargetScan, miRDB, and RNAInter databases, which revealed three genes (UCP2, THY1, errfi1) from intersection analysis (Figure 3B). Among the three, THY1 has been reported previously to inhibit IPF [11], and was therefore selected as the main research object. The possible binding site of miR-214 on THY1 was predicted by TargetScan database (Figure 3C). To validate this predicted binding relationship, dual-luciferase reporter assay was conducted to detect the luciferase activity of Wt-THY1-3’UTR and Mut-THY1-3’UTR that respectively co-transfected with agomir NC and miR-214 agomir in HEPFs (Figure 3D). The results showed that miR-214 could specifically target THY1 and down-regulate the luciferase activity of Wt-THY1-3’UTR. Therefore, we speculated that miR-214 might inhibit THY1 to mediate pulmonary fibrosis, and carried out further experiments to test this hypothesis.

We silenced THY1 in HEPFs using siRNAs and inhibited miR-214 expression using miR-214 antagomir. si-THY1-3 with the best silencing efficiency was determined by RT-qPCR and western blot analysis, and was selected for subsequent experiments (Figure 3E). The expression of miR-214 and THY1 mRNA in HEPFs was measured by RT-qPCR, and protein expression of THY1 was assessed by western blot analysis (Figure 3F), showing that the silencing efficiency was sufficient for further experiments. In addition, treatment with miR-214 antagomir led to up-regulation of THY1 expression, indicating that miR-214 negatively regulated THY1 expression. The viability of HEPFs in response to treatment with si-THY1 and/or miR-214 antagomir was measured by CCK8 experiments (Figure 3G). The results revealed that silencing miR-214 could inhibit the viability of HEPFs, while the inhibition of THY1 simultaneously could reverse this inhibitory effect. Furthermore, the expression of cell proliferation marker Ki67 and fibrosis biomarkers CoI, CoIII, α-SMA and vimentin was measured by RT-qPCR and western blot analysis (Figure 3H), which showed that silencing miR-214 reduced the expression of Ki67 and that of fibrosis biomarkers, while silencing THY1 could neutralize this effect. To sum up, miR-214 could down-regulate the expression of THY1, and silencing miR-214 could inhibit the process of HEPFs differentiation into myofibroblasts by increasing THY1.

### Involvement of YY1/HSF1/miR-214/THY1 axis in the proliferation and fibrogenic transformation of HEPFs

**Figure 4:**
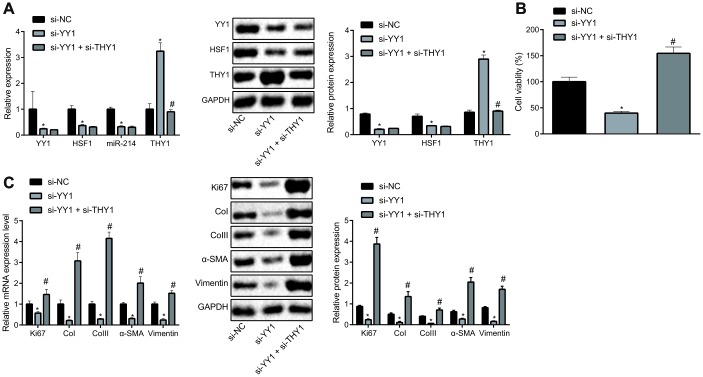
**YY1/HSF1/miR-214/THY1 regulates the proliferation and fibrogenic transformation of HEPFs.** (**A**) The expression of YY1, HSF1, miR-214 and THY1 determined by RT-qPCR, and the expression of YY1, HSF1, and THY1 proteins measured by western blot analysis in the HEPFs transfected with si-NC, si-YY1 or si-YY1 and si-THY1 in combination; (**B**) The cell viability assessed by CCK8 after transfection with si-NC, si-YY1 or si-YY1 and si-THY1 in combination; (**C**) The mRNA and protein expression of cell proliferation marker Ki67 and fibrosis biomarkers CoI, CoIII, α-SMA and vimentin in the HEPFs transfected with si-NC, si-YY1 or si-YY1 and si-THY1 in combination measured by RT-qPCR and western blot analysis. Statistical data were measurement data, and presented as mean ± standard deviation. Unpaired *t*-test was used for comparison between the two groups, and one-way ANOVA with Tukey's *post hoc* test was employed for comparisons among multiple groups. *, *p* < 0.05 *vs.* cells transfected with si-NC, #, *p* < 0.05 *vs.* cells transfected with si-YY1. The experiment was independently repeated three times.

Based on the aforementioned findings, we attempted to verify the effect of the YY1/HSF1/miR-214/THY1 axis on the fibrogenic transformation of HEPFs. After silencing YY1 alone or together with THY1 in HEPFs, expression of YY1, HSF1, miR-214 and THY1 was assessed by RT-qPCR, and the protein expression was measured by western blot analysis (Figure 4A). The results showed that, compared with the cells transfected with si-NC, the expression of YY1, HSF1 and miR-214 in cells transfected with si-YY1 was significantly decreased, while the expression of THY1 was noticeably increased. Meanwhile, expression of YY1, HSF1 and miR-214 showed no significant change, but the expression of THY1 was notably reduced in cells treated with si-YY1 and si-THY1 compared with the cells transfected with si-YY1. Hence, YY1 could down-regulate the expression of THY1. Then, the viability of HEPFs in response to YY1 silencing alone or in combination with THY1 was measured by CCK8 experiments (Figure 4B). Results suggested that silencing YY1 could inhibit the viability of HEPFs, which was rescued by silencing THY1 simultaneously. Additional RT-qPCR and western blot analysis data (Figure 4C) revealed that silencing YY1 caused reductions in the expression of Ki67 and that of fibrosis biomarkers, while silencing THY1 could attenuate hose reductions. These results showed that the proliferation and fibrogenic transformation processes of HEPFs could be mediated through YY1/HSF1/miR-214/THY1 axis.

### YY1 silencing can down-regulate to THY1 to slow down the progression of IPF in mice

**Figure 5:**
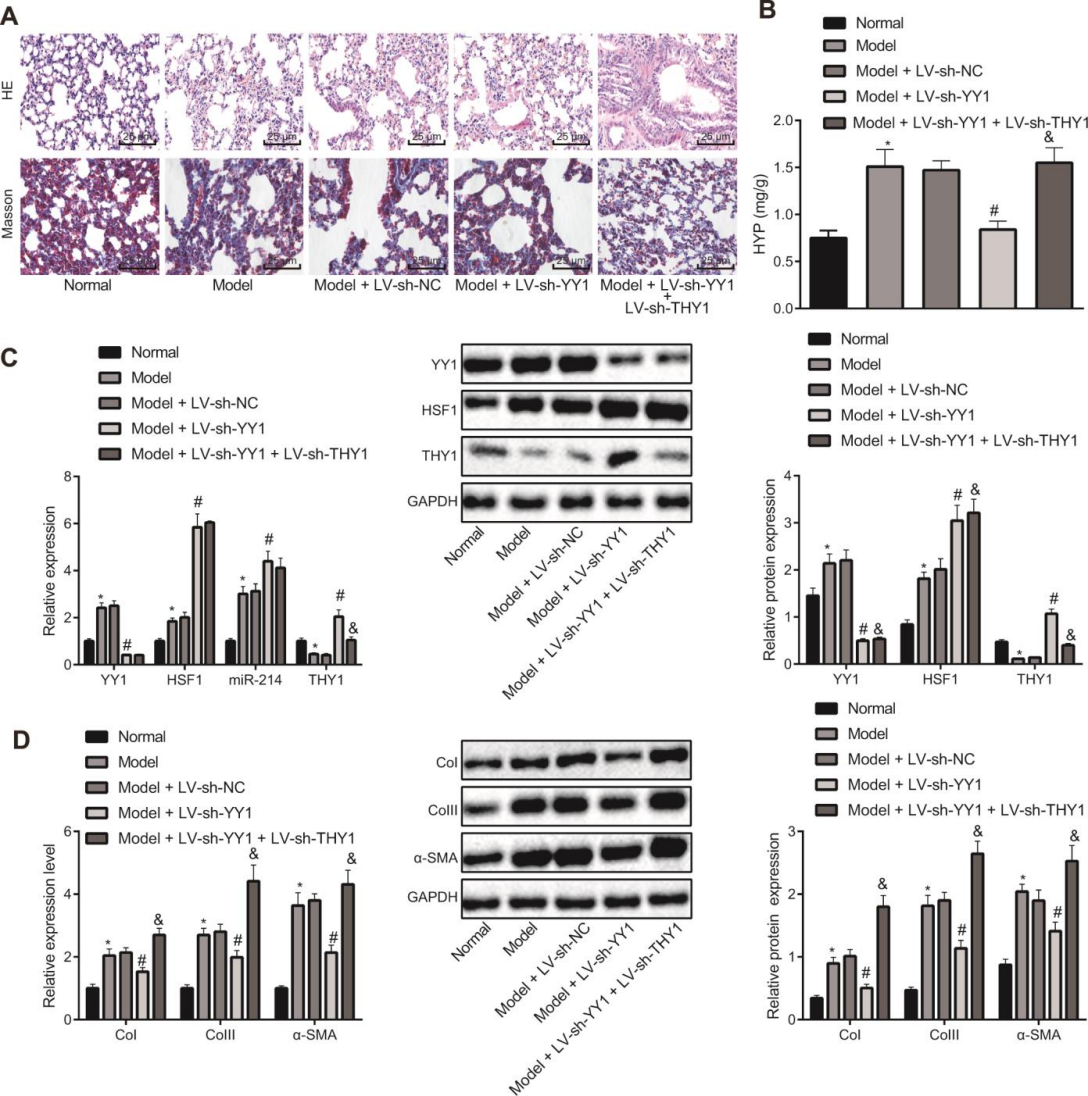
**Effect of YY1/HSF1/miR-214/THY1 axis on bleomycin-induced IPF in mice.** To generate a IPF model, the mice were administrated with bleomycin dissolved in normal saline on the 1^st^, 5^th^, 8^th^, 11^th^ and 15^th^ days, and infected with lentivirus (LV-sh-NC, LV-sh-YY1, LV-sh-YY1 and LV-sh-THY1 in combination) on the 24^th^, 26^th^ and 28^th^ days. (**A**) The pathological changes in lung tissues in mice shown by HE staining and Masson staining, respectively (400×); (**B**) The amount of collagen in lung tissues shown by hydroxyproline (HYP) measurement using alkaline hydrolysis assay; (**C**) The expression of YY1, HSF1, miR-214 and THY1 in lung tissues of mice measured by RT-qPCR, and the expression of YY1, HSF1 and THY1 proteins measured by western blot assay; (**D**) The expression of COI, COIII and α-SMA in the lung tissues of mice measured by RT-qPCR and western blot assay. Statistical data were measurement data, and presented as mean ± standard deviation. Comparisons among multiple groups were analyzed by the one-way ANOVA with Tukey's *post hoc* test. *, *p* < 0.05 *vs.* control mice, #, *p* < 0.05 *vs.* bleomycin-treated mice injected with LV-sh-NC, &, *p* < 0.05 *vs.* bleomycin-treated mice injected with LV-sh-YY1, n = 8.

To further verify the implications of the YY1/HSF1/miR-214/THY1 axis in IPF development, we used bleomycin to induce a mouse pulmonary fibrosis model. The model mice were injected with the lentivirus-packaged plasmids LV-sh-THY1, LV-sh-YY1 alone or in combination. HE staining and Masson staining were used to detect the pathological changes of lung tissues in these mice (Figure 5A and Table 1). Compared with control mice, the bleomycin-treated mice had obvious inflammatory cell infiltration in the alveolar septum and interstitium, thickening of alveolar septa, capillary hyperplasia, extensive lesions, large area of collagen fiber bundle, thickening of alveolar wall, obvious increase of collagen deposition in bronchi and blood vessels, and increased fibrosis score. The infection with LV-sh-YY1 contributed to improved lung pathological manifestations in the bleomycin model mice, while LV-sh-THY1 treatment worsened the lung pathological manifestations, which were alleviated by LV-sh-YY1 (*P* < 0.05). Then hydroxyproline measurement by alkaline hydrolysis was employed to measure the amount of collagen in lung tissues (Figure 5B), results of which were consistent with the Masson staining.

**Table 1:** Score for Pulmonary inflammation and fibrosis.

**Group**	**n**	**Inflammatory scores**				**Fibrosis scores**			
		**0**	**1**	**2**	**3**	**0**	**1**	**2**	**3**
Normal	8	8	0	0	0	8	0	0	0
Model^*^	8	0	0	3	5	0	0	2	6
Model+LV-sh-NC	8	0	0	3	5	0	0	2	6
Model+LV-sh-YY1^#^	8	0	4	3	1	0	2	3	3
Model+LV-sh-YY1+LV-sh-THY1^&^	8	0	0	1	7	0	0	1	7

RT-qPCR and western blot analyses (Figure 5C) suggested that compared with control mice, mRNA expression of YY1 and HSF1, and expression of miR-214 in the mice with bleomycin-induced pulmonary fibrosis were significantly increased and the expression of THY1 was notably decreased (*P* < 0.05). Furthermore, the expression of YY1 in the bleomycin-treated mice injected with LV-sh-YY1 was reduced, and consequently the expression of HSF1, miR-214 and THY1 was elevated by knocking down YY1 (*P* <0.05). Compared with the mice injected with LV-sh-YY1, the expression of YY1, HSF1 and miR-214 in model mice after treatment of LV-sh-YY1 and LV-sh-THY1 showed no significant change (*P* > 0.05), but the expression of THY1 was decreased significantly (*P* < 0.05).

Finally, we measured the expression of CoI, CoIII and α-SMA in the lung tissues of model mice by RT-qPCR and western blot analysis (Figure 5D). Results showed that, compared with normal control mice, the expression of CoI, CoIII and α-SMA in the bleomycin-treated mice was significantly elevated (*P* < 0.05). The expression of CoI, CoIII and α-SMA in lung tissues of the bleomycin-treated mice was notably reduced by infection with LV-sh-YY1 (*P* <0.05). Compared with mice injected with LV-sh-YY1, the expression of CoI, CoIII and α-SMA in lung tissues was significantly elevated after treatment of LV-sh-YY1 and LV-sh-THY1 (*P* < 0.05).

## DISCUSSION

A growing body of evidence has suggested that IPF is an epithelial-mediated disease, where activated lung epithelial cells initiate fibroblast migration and proliferation, and fibroblast-to-myofibroblast transition [13]. Activation of myofibroblasts results in accumulation of extracellular matrix, which itself leads to remodeling of the lung architecture [13]. A better understanding of the pathogenesis of this disease will help to identify novel and effective therapeutic approaches.

Previous studies have demonstrated that YY1 is up-regulated in lung fibroblasts induced by transforming growth factor-β (TGF-β) or tumor necrosis factor-α (TNF-α) [4], both of which are central pro-fibrotic mediators of pulmonary fibrosis [14, 15]. Consistent with this, we observed up-regulation of YY1 in mice with bleomycin-induced IPF. Using loss-of-function assays, silencing YY1 was shown to decrease the expression of proliferation and fibrosis markers, suggesting that this process could repress proliferation and fibrogenic transformation of fibroblasts. Furthermore, we found that YY1 could positively regulate the expression of HSF-1, which has also been reported in a previous study [16]. Using rescue assays, we demonstrated that overexpression of HSF-1 counteracted the effects achieved by YY1 silencing. Hence, silencing YY1 resulted in down-regulation of HSF-1, and consequently led to suppression of fibroblast proliferation and fibrogenic transformation. In another study focusing on collagen production, HSF-1 overexpression has been validated to restore the collagen production otherwise restrained by TRAIL treatment [17], indicative of its pro-fibrotic potential. The present study also suggested a pro-fibrotic role of HSF-1 in lung fibroblasts. Moreover, we found that inhibition of HSF-1 contributed to an anti-fibrotic mechanism by suppressing proliferation and fibrogenic transformation of lung fibroblasts.

Accumulating evidence has suggested that miR-214 exaggerates IPF [9, 18]. Moreover, a recent study has also indicated that the transcription of miR-214 is modulated by HSF-1 in neurons [8]. Thus, we asked if this regulatory mechanism also occurs during the pathogenesis of IPF. Our study showed that ablation of HSF-1 resulted in down-regulation of miR-214. More importantly, the suppressive effects of HSF-1 silencing on fibroblast proliferation and fibrogenic transformation were reversed by further overexpression of miR-214. This finding showed that down-regulation of miR-214 caused by si-HSF-1 may be responsible for the anti-fibrotic effect of HSF-1 silencing. The pro-fibrotic effect of miR-214 has also been addressed in several studies in other organs such as liver and kidney [19, 20]. More recently, miR-214 has been identified as a downstream molecule of lncRNA FENDRR, and shown to have a pro-fibrotic action in lung fibroblasts [18]. In the present study, we further investigated the downstream molecular events of miR-214 through online prediction servers (TargetScan, miRDB, and RNAInter). We also analyzed the dataset (GSE24206), which revealed the DEGs between healthy individuals and IPF patients [21]. Among those DEGs and putative target genes, we identified THY-1 as a potential target of miR-214, and then confirmed their binding relationship. Interestingly, THY-1 has already been considered as an anti-fibrosis molecule, which can attenuate IPF [11, 12]. Also, its ablation may confer a profibrotic phenotype of lung fibroblasts [22]. Notably, our new data revealed that inhibiting miR-214 significantly up-regulated the expression of THY-1, by which treatment with miR-214 antagomir reduced the proliferation and fibrogenic transformation of fibroblasts. The above findings support our hypothesis that the YY1-HSF-1-miR-214-THY-1 axis functions as a signaling cascade to regulate the pathogenesis of IPF.

To further confirm this hypothesis, we used loss-of-function analysis, which showed that YY1 ablation resulted in down-regulation of HSF-1 and miR-214, and up-regulated THY-1. As expected, when we knocked down both YY1 and THY-1 in lung fibroblasts, the decreased cell proliferation and fibrosis marker expression otherwise induced by YY1 silencing was compromised. These observations indicate that the YY1-HSF-1-miR-214-THY-1 axis could indeed function in regulating the pathogenesis of IPF, which was validated in the murine model of experimental IPF. Notably, other mechanisms have also been proposed to interpret the pathogenesis of fibrosis. For example, HSF-1 has been reported to promote cardiac fibrosis by down-regulating the expression of Smad family member 3 (Smad3) [23]. Moreover, miR-214 has been shown to contribute to cardiac remodeling by mediating cardiac fibroblast proliferation in an ERK1/2 dependent manner [24]. These findings indicate that the signaling pathway regulating the fibrosis may be cell type specific.

**Figure 6:**
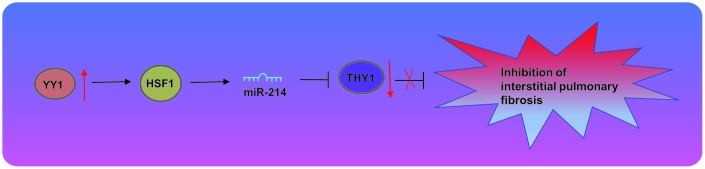
**Transcription factor YY1 inhibits the expression of THY1 to promote interstitial pulmonary fibrosis by activating the HSF1/miR-214 axis.**

In conclusion, our study has identified a novel modulatory axis, YY1-HSF-1-miR-214-THY-1, and defined the underlying mechanism by which YY1 promotes the pathogenesis of IPF (Figure 6). Our study has furthered the understanding of the pathophysiology of IPF and presents a potential therapeutic target for this disease. However, the translation of those finding into clinics warrants further substantiation in a large-sample clinical trial.

## MATERIALS AND METHODS

### Ethics statement

Animals were treated humanely using approved procedures in compliance with the recommendations in the Guide for the Care and Use of Laboratory Animals of the National Institutes of Health. The protocol was approved by the Institutional Animal Care and Use Committee of Sichuan Academy of Medical Sciences and Sichuan Provincial People's Hospital, University of Electronic Science and Technology of China.

### *In silico* analysis

IPF-related microarray GSE22406 was downloaded from Gene Expression Omnibus (GEO) database (https://www.ncbi.nlm.nih.gov/), including 6 samples of normal controls and 17 samples of IPF. DEGs were screened with |LogFoldChange| > 1 and *p-v*alue < 0.05 as the screening criteria. The target genes of miR-214 were predicted by TargetScan database (http://www.targetscan.org/vert_71/), StarBase database (http://starbase.sysu.edu.cn/) and RNAIner database (http://www.rna-society.org/rnainter/). Due to different algorithms and thresholds of the three databases, the putative targets obtained from the three databases and the DEGs in GSE22406 microarray were intersected to predict the target gene of miR-214 that related to IPF.

### Cell treatment

HEPFs were purchased from Shanghai Institute of Biochemistry and Cell Biology (Shanghai, China). The cells were cultured in RPMI 1640 medium (Sigma-Aldrich Chemical Company, St Louis, MO, USA) with 10% serum (Invitrogen Inc., Carlsbad, CA, USA) at 37°C and 5% CO_2_. The medium was renewed every two days, and upon reaching 80% confluence, cells were passaged at a ratio of 1:2.

The passaged cells were treated with the plasmids of si-YY1, oe-HSF1, si-HSF1, miR-214 agomir, miR-214 antagomir, si-THY1, or their negative controls (NCs) alone or in combination. The overexpression vector pcDNA3.1, agomir, antagomir and sequence of siRNA were designed and constructed by GenePharma Co., Ltd. (Shanghai, China). The transfection was conducted under the instructions of Lipofectamine 2000 kit (Invitrogen).

### RNA isolation and quantitation

Total RNA was extracted by RNeasy Mini kit (Qiagen company, Hilden, Germany). The mRNA was reversely transcribed into complementary DNA (cDNA) by the reverse transcription kit (RR047A, Takara Bio Inc., Otsu, Shiga, Japan). And miRNA was reversely transcribed into cDNA with the miRNA First Strand cDNA Synthesis (Tailing Reaction) kit (B532451-0020, Shanghai Sangon Biotechnology Co. Ltd., Shanghai, China). Fluorescence PCR was performed according to the instructions of SYBR^®^ Premix ExTaq^TM^ II kit (DRR081, Takara Bio Inc.) on a real-time PCR (ABI 7500, ABI, Shanghai, Foster City, CA, USA). Each sample was tested in triplicate. The universal negative primer of miRNA and the U6 upstream primer of human and mouse were provided by the miRNA First Strand cDNA Synthesis (Tailing Reaction) kit, and the other primers applied universally for human and mouse were synthesized by Sangon (Table 2). GAPDH or U6 was regarded as the internal reference, and the relative expression of each target gene was calculated by 2^-ΔΔCt^ method.

**Table 2:** The primer sequences for RT-qPCR.

**Genes**	**Sequences**
YY1	F:5’-AAGAGCGGCAAGAAGAGTTAC-3’
	R:5’-CAACCACTGTCTCATGGTCAATA-3’
HSF1	F:5’-CCATGAAGCATGAGAATGAGGC-3’
	R:5’-CTTGTTGACGACTTTCTGTTGC-3’
miR-214	F:5’-ACAGCAGGCACAGACAGGCAGT-3’
THY1	F:5’-ATCGCTCTCCTGCTAACAGTC-3’
	R:5’-CTCGTACTGGATGGGTGAACT-3’
Ki67	F:5’-ACGCCTGGTTACTATCAAAAGG -3’
	R:5’-CAGACCCATTTACTTGTGTTGGA-3’
CoI	F:5’-TCTGACTGGAAGAGTGGAGAGTAC-3’
	R:5’-ATCCATCGGTCATGCTCTCG-3’
CoIII	F:5’-GAATGGTGGCTTTCAGTTCAGC-3’
	R:5’-GCTGTTTTTGCAGTGGTATGTAATG-3’
α-SMA	F:5’-TCAAATACCCCATTGAACACGG-3’
	R:5’-GGTGCTCTTCAGGTGCTACA-3’
Vimentin	F:5’-TGCGTGAAATGGAAGAGAACT-3’
	R:5’-TGCGTGAAATGGAAGAGAACT-3’
GAPDH	F:5’-GGATTTGGTCGTATTGGG-3’
	R:5’-GGAAGATGGTGATGGGATT-3’

Note: RT-qPCR, reverse transcription quantitative polymerase chain reaction; YY1, Yin Yang 1; HSF1, Heat Shock Transcription Factor 1; α-SMA, Actin alpha, smooth muscle aorta; GAPDH, glyceraldehyde-3-phosphate dehydrogenase; F, forward; R, reverse.

### Western blot analysis

Total protein was extracted by Radio Immunoprecipitation Assay (RIPA) lysis buffer with phenylmethylsulfonyl fluoride (PMSF), and incubated on ice for 30 min, followed by centrifugation at 4°C, 8000 g × for 10 min. The bicinchoninic acid (BCA) kit was used to measure the total protein concentration in the supernatant. The protein (50 μg) was separated by electrophoresis and transferred onto a polyvinylidene fluoride (PVDF) membrane. After being blocked with 5% skim milk for 1 h, the PVDF membrane was incubated with diluted anti rabbit YY1 antibody (ab109228, 1:1000), HSF1 antibody (ab2923, 1:1000), THY1 antibody (13801s, 1:1000, Cell Signaling technology, Beverly, MA, USA), Ki67 antibody (ab16667, 1:1000), COI antibody (ab210785, 1:1000), COIII antibody (ab7778, 1:5000), α-SMA antibody (ab32575, 1:1000), vimentin antibody (ab92547, 1:1000) overnight at 4°C, with GAPDH (ab181602, 1:10,000) regarded as internal reference. Subsequently, the membrane was incubated with horseradish peroxidase (HRP) conjugated secondary antibody goat anti-rabbit IgG H&L (ab97051, 1:2000) for 1 h. All the above antibodies except THY1 were purchased from Abcam Inc. (Cambridge, UK). Then, equal portions of solutions A and B were taken from the enhanced chemiluminescence (ECL) kit (product No. bb-3501, Amersham Ltd., UK), and added to the membrane. The image was obtained using Bio-Rad gel imaging system (Bio-Rad Laboratories, Hercules, CA, USA). Quantity One v4.6.2 software was used for quantitative analysis. The ratio of corresponding protein bands to internal reference GAPDH gray values indicated the corresponding protein content.

### Dual-luciferase reporter assay

The wild type and mutant pmirRGLO luciferase reporter plasmids (pGL3-Wt-THY1-3’UTR, pGL3-Mut-THY1-3’UTR) of THY1 were designed by GenePharma Co., Ltd.. The agomir NC and miR-138 agomir were co-transfected with Wt-THY1-3’UTR and Mut-THY1-3’UTR respectively into HEPF. After 48 h of transfection, the cells were collected and lysed following the instructions of the luciferase detection kit (k801-200, BioVision Technologies, Inc., Bioptics, Tucson, USA). Activation of the target reporter gene was analyzed by dual-luciferase reporter gene system (Promega Corporation, Madison, WI, USA).

### Cell counting kit 8 (CCK-8)

Cell counting kit-8 (Kumamoto, Japan) was utilized to assess cell viability. In brief, the cells were seeded into a 96-well plate. After 48 h of transfection, CCK-8 reagent (10 ul) was added to each well and further incubated with the cells for 1 h. Finally, the absorbance at the wavelength of 450 nm was evaluated by microplate reader.

### A pulmonary fibrosis mouse model and lentivirus injection

Forty C57BL/6J mice (aged 8 weeks; weighing 20-25 g) purchased from the experimental animal research institute of Sichuan Academy of Medical Sciences were raised under standard conditions. The mice were grouped into 5 sets of 8 mice each: mice treated with bleomycin, bleomycin-treated mice infected with LV-sh-NC, LV-sh-YY1, LV-sh-YY1 and LV-sh-THY1 in combination, and normal control mice. The recombinant lentivirus was designed and constructed by Hanbio Biotechnology Co., Ltd. (Shanghai, China). On the 1^st^, 5^th^, 8^th^, 11^th^ and 15^th^ day, bleomycin dissolved in normal saline was intraperitoneally injected into each mouse at a dose of 50 mg/kg. The lentivirus was intraperitoneally injected into the mice at a dose of 2 × 10^6^ transducing units (TU) on the 24^th^, 26^th^ and 28^th^ day, respectively. Mice were euthanized by carbon dioxide method on the 40^th^ day, and the lung tissues were taken for RNA and histological analysis.

### Histopathological analysis

The upper lobe of the right lung was fixed with 10% formalin solution and embedded in paraffin, whereupon the sections were stained with hematoxylin, eosin and Masson. The numerical inflammation and fibrosis scoring scales (Tables 3 and 4) were used for quantitative histological analysis. The mean of the two scores was considered to be the inflammation and fibrosis score.

**Table 3:** Score for alveolar inflammation.

**Score**	**Description**
0	No lung tissue inflammation
1	Mild pulmonary inflammation, inflammatory cell infiltration is limited to local or near the pleura, the area is less than 20% of the whole lung
2	Moderated pulmonary tissue inflammation, the affected area of the lung accounted for 20% to 50%
3	Severe pulmonary tissue inflammation, involved area was greater than 50%

**Table 4:** Score for pulmonary fibrosis.

**Score**	**Description**
0	No pulmonary fibrosis
1	Mild pulmonary fibrosis, the affected area less than 20% of the whole lung
2	Moderated pulmonary fibrosis, the affected area of the lung accounted for 20% to 50%
3	Severe pulmonary fibrosis, involved area was greater than 50%

### Alkaline hydrolysis

A total amount of 40-80 mg lung tissues was mixed with 1 ml hydrolase and bathed in boiling water for 20 min. The supernatant was centrifuged at 3500 rpm for 10 min according to the instructions of hydroxyproline (Hyp) test kit (xfa030-2, Shanghai Biorui Biotechnology Co., Ltd., Shanghai, China). The absorbance at 550 nm was measured by spectrophotometer, and the Hyp content in the lung tissues of each mouse was calculated.

### Statistical analysis

All data were processed by SPSS 22.0 software (SPSS, IBM Corp., Armonk, NY, USA). Measurement data was expressed as mean ± standard deviation. Unpaired *t*-test was used to compare the data between two groups and comparisons among multiple groups were analyzed by unpaired one-way analysis of variance (ANOVA) with Tukey's *post hoc* test. A value of *p* < 0.05 was considered to be statistically significant.
